# Measuring self-reliance and wellbeing among forcibly displaced women enrolled in a gender-focused economic empowerment program: A protocol for a pilot randomized controlled trial in Colombia

**DOI:** 10.1371/journal.pone.0318224

**Published:** 2025-01-27

**Authors:** Lindsay Stark, Ilana Seff, Adriana Monar, Diany Castellar, Neetu Mahi, Arturo Harker Roa

**Affiliations:** 1 Brown School at Washington University in St. Louis, St. Louis, Missouri, United States of America; 2 HIAS, Washington, DC, United States of America; 3 Universidad de los Andes, Bogota, Colombia; IFPRI: International Food Policy Research Institute, UNITED STATES OF AMERICA

## Abstract

Colombia currently hosts nearly three million Venezuelan refugees and migrants and is home to seven million internally displaced Colombians. For forcibly displaced populations in Colombia, and especially for women, gender-based violence (GBV) poses a threat during transit and in their new homes where xenophobia, lack of accessible and adequate services, limited safe economic opportunities, and lack of information on access to services, further increase risk. The dearth of livelihood opportunities also affects forcibly displaced populations, especially women. This study will conduct a randomized-controlled trial (RCT) of HIAS’ Entrepreneurship School with Gender Lens (ESGL), a program that targets forcibly displaced GBV survivors and women at-risk to help them develop business ideas, access needed support for the prevention of and response to GBV, and improve overall self-reliance. The RCT will be conducted within three cities in Colombia; approximately 80 eligible participants will be enrolled in each city and randomized to a treatment or control arm. Survey questionnaires will be administered to participants at baseline, eight months following baseline (endline), and 3–4 months after endline. Outcomes of interest include household self-reliance, mental health, resilience, empowerment, utilization of GBV services, and social support.

## Background

Colombia is home to more than ten million forcibly displaced persons, including 6.9 million internally displaced peoples (IDPs) and three million refugees and migrants from Venezuela [1]. The psychosocial impacts of the migratory experience, including depression and anxiety, are well-documented and, due to the confluence of xenophobia, a lack of accessible and adequate services and safe economic opportunities, and inequitable gender norms, forcibly displaced people in Colombia face elevated risks of gender-based violence (GBV) [2–4]. Experiences of GBV may further erode mental health and well-being, with survivors more likely to exhibit low self-esteem, depression, fear, anguish, guilt, and post-traumatic stress [5]. In Colombia, evidence demonstrates that limited availability of shelters and a lack of protection services in border areas place forcibly displaced women at a higher risk of violence by organised crime and drug traffickers [6]. Young women and girl migrants are also vulnerable to sexual exploitation, which further perpetuates cycles of violence and other negative consequences such as early pregnancy [7].

A dearth of livelihood opportunities for forcibly displaced populations, and particularly women, exacerbates these vulnerabilities [8]. A recent Labor Market and Capacity Assessment in Colombia [9] identified numerous barriers to seeking safe employment, including barriers in terms of regularisation; lack of information on job opportunities; precarious working conditions with lower payments, longer working days and increasing risks of labor exploitation; xenophobia and discrimination; limited access to formal labor markets; lack of access to financial services, and others. The lack of economic empowerment, together with widespread gender discrimination and the reinforcement of traditional stereotypes, affects the ability of forcibly displaced women to participate equitably in many aspects of public and private life [10].

By fostering personal choice and human agency, scholars have shown that economic empowerment programs can improve overall individual psychosocial wellbeing [11,12]. More specifically, poverty alleviation and finance programs targeted towards women in low-income countries can work to reduce impoverishment, increase access to resources, and enhance empowerment [13–15]. Women’s increased share of income within the household may also usher in greater negotiating power and decision-making capabilities, thereby reducing experiences of gender-based violence [16,17]. At the same time, some research supports the male backlash theory, which posits that men may use violence when their traditional position of power is threatened by their female partner’s newer access to economic resources [18,19]. Despite some evidence supporting this theory in settings that adhere to strict patriarchal norms, research has identified women’s entrepreneurship as a critical avenue for women’s empowerment, allowing women entrepreneurs to create jobs, drive economic growth, and enact a significant impact on their communities’ economic and social growth [20,21].

Overall, research abounds on the effectiveness of women’s entrepreneurial programming in fostering women’s economic empowerment and self-sufficiency, yet gaps persist in understanding these program’s impact on women’s mental health and their households’ self-reliance, as well as understanding how these impacts manifest for forcibly displaced populations, specifically. This study will conduct a pilot randomised-controlled trial to evaluate the Entrepreneurship School with Gender Lens (ESGL), an entrepreneurial program that targets forcibly displaced women who have experienced GBV or are at risk of GBV to help them develop business ideas, access resources, and improve overall self-reliance. This program follows a methodology that allows entrepreneurs to transform their business idea into a business plan for further implementation, while incorporating a specific gender lens to promote economic sufficiency for women. Ultimately, the evaluation aims to (1) evaluate the preliminary effectiveness of the entrepreneurship programming to improve household self-reliance, mental health, and empowerment for forcibly displaced households where women members run businesses; (2) examine the relationship between individual-level mental health, resilience, and social support and household-level self-reliance among forcibly displaced women in the Colombian migration context; and (3) assess the feasibility and acceptability of the Self-Reliance Index (SRI) as a measurement tool in programs that aim to strengthen self-reliance pathways for women individuals.

## Methods/Design

This pilot RCT will be conducted in three cities in Colombia: Cali, Pasto, and Popayan. Eligible participants will be randomized to the treatment or control arm and the study team will collect data from both the intervention and control groups at: 1) the program baseline, 2) the program endline (8 months post-baseline), and 3) follow-up 3–4 months after program completion.

### Study setting

This pilot RCT will take place in three Colombian cities—Cali, Pasto, and Popayan—where Venezuelan migrants have sought shelter from economic hardship and political instability [22]. Colombia has made sizable progress towards welcoming Venezuelan migrants and fostering self-resiliency, most notably by instituting a mass regularization program to grant legal status and work authorization to this population. However, with a new government and legislation that has reduced support services for forcibly displaced Venezuelans, progress on economic inclusion and integration has drastically decreased and many migrants lack access to food, shelter, healthcare, education, and formal employment [23]. The Colombian government has also stopped coordinating humanitarian actors and NGOs, reducing non-governmental support for refugees and migrants [24]. This evolving situation necessitates further response and investment for the refugee populations within Colombia.

These three cities also serve as hubs for internally displaced Colombians. Colombia has one of the largest internally displaced people (IDP) populations in the world at 6.8 million [23]. In 2013, one-in-eight Colombian citizens were displaced [25]. With the demobilization of the Revolutionary Armed Forces of Colombia (FARC) and the COVID-19 pandemic, dissidents of the peace process and other non-State armed groups have fought for territorial and social control, leading to increases in forced displacement, confinements of communities, extortion, forced recruitment, and targeted violence [23,25]. Vulnerable populations primarily consist of campesinos, indigenous peoples, and Afro-Colombians, whose lands have most often been confiscated forcing these groups to migrate from rural to urban areas. As such, IDP populations are mainly made up of women, children, and ethnic minorities [25].

### Intervention description

Hebrew Immigrant Aid Society (HIAS) is a non-profit organization that provides humanitarian aid and assistance to refugees, asylum seekers and other forcibly displaced populations around the world. HIAS will promote the incubation of businesses through its nine-month long Entrepreneurship School with Gender Lens (ESGL) intervention, which targets forcibly displaced women who have experienced GBV or are at risk of GBV to help them develop business ideas, access needed support for the prevention of and response to GBV and improve participants’ households’ self-reliance. The business training stage includes seven four-hour group training sessions facilitated by Economic Inclusion Officers (See Table 1) and one individual counseling session facilitated by mental health and psychosocial support (MHPSS) staff. These sessions transversally integrate topics of MHPSS and GBV prevention, mitigation and response, empowerment, and protection. At the end of the training, an additional preparatory session is held in preparation for the presentation of the business plan to the evaluation committee.

**Table 1:** ESGL program modules.

SESSION	Business topics	GBV/MHPSS topics
**Session 1**		Autonomy and empowerment through a gender lens
**Session 2**	Motivations to develop my business idea	Empowerment, gender-related barriers, rights, motivation, women in entrepreneurship, socio-emotional skills
**Session 3**	Market contextualization for the development of innovative products	Importance and the role of women in business, challenges faced by women in business
**Session 4**	Resource management and conflict resolution in the workplace	Conflict management, resilience Emotion management
**Session 5**	Willpower associated with business	Women’s economic empowerment and autonomy
**Session 6**	Analysis for financial decision making	
**Session 7**	Building the business plan	What to do when facing GBV risks?

The training methodology applies a pedagogical approach that facilitates the construction of concepts based on participants’ prior knowledge, and the construction of new knowledge and skills contextualized to the experience and life of the participating women.

Upon training completion, participants are eligible to pitch their business idea to a panel of experts and receive up to USD$800 of seed capital. Participants also receive business advisory support for at least six months, focusing on access to markets and building support networks. HIAS Economic Inclusion Officers work on establishing goals with participants and track, adjust and revise goals during the follow-up sessions. These sessions also provide the opportunity to assess protection risks and offer additional referrals, as needed.,It is hypothesized that building these skills while also strengthening participants’ ability to engage in dignified livelihoods will lead to improvements in mental health, psychosocial wellbeing, and self-reliance.

### Study population, recruitment, and retention

Eligibility criteria for this study include: having ever experienced or being at risk of GBV; being an adult of 18 years or older; have lived in Colombia for at least six months; are Venezuelan or Colombian; have already shared an entrepreneur profile with HIAS; and score within a predetermined range on the SRI. Eligibility criteria will be assessed across three stages: pre-targeting, pre-screening, and screening.

In the pre-targeting phase, HIAS will identify potential participants through organizational databases, referrals from other organizations, and calls to the community. The objective of the pre-targeting stage is to identify women who may be at risk of or who have experienced GBV. To identify women meeting this criteria, HIAS program staff will employ an instrument that flags relevant risk factors, including female headed households, access to resources and autonomy, and pregnancy and breastfeeding. Additionally, it identifies whether women are GBV survivors, have ever experienced incidents of GBV, or have ever accessed GBV response services. Given that many women find it difficult to recognize GBV, a definition of GBV will be provided to ensure clarity when questions are asked.

Women who are identified as experiencing GBV will be referred to HIAS GBV Case Management services if they consent. Forms for risk characterization and assessment will also be applied within the framework of those services. Using that information, case workers will build action and safety plans according to HIAS global standards and international GBV case management guidelines.

In the pre-screening phase, HIAS will pre-screen approximately 90–100 pre-targeted women per city using the pre-screening tool. Eligibility criteria assessed during this stage include: being an adult of 18 years or older; have lived in Colombia for at least six months; are Venezuelan or Colombian; and have already shared an entrepreneur profile with HIAS.

Official enrollment, consenting, and formal assessment of eligibility will be carried out by the research team. Data collectors will obtain written consent from previously pre-targeted and pre-screened women prior to administering the SRI. Before obtaining written consent, data collectors will explain the purpose of the study, discuss any benefits or risks to participation and safeguarding procedures, and answer any questions the potential participant may have. Data collectors will explain that participation in the study is voluntary and that participants may decide to leave the study at any time, without penalty or consequence.

After administering the SRI, data collectors will immediately administer the remaining survey questionnaire modules for participants whose SRI scores fall within the eligible range of 2 to 4.25 (the final eligibility criterion). This range of self-reliance (measured on a scale from 1 to 5; please see more on the SRI, below) was selected as an appropriate range for target participants for two reasons: (i) Those who score below a ‘2’ are in such dire circumstances that they are unlikely to be able to capitalize on an entrepreneurial program, and (ii) those who score above a 4.25 have achieved a level of self-reliance that may not leave substantial room for growth. Women whose scores fall outside of the 2–4 range will be thanked for their time and not assigned to an evaluation arm.

Participants will be recruited from three cities: Cali, Pasto, and Popayan. Recruitment is scheduled to start in July 2024. The study will aim to enroll at least 80 women in each city. where participants in each city will be randomly assigned to the intervention or control arm using a random number generator. The number of participants randomized to the treatment and control arm within each city will be determined by the number of women HIAS has the capacity to support in the intervention. For example, if HIAS can (and is required to) support 50 women in the intervention in one city, 50 women will be randomly assigned to the treatment arm and 30 to the control arm.

At the time of enrollment, participants will receive a unique study ID. The unique study ID will be used to track participants over time for future points of data collection.

### Study timeline, data collection and outcomes of interest

#### Data collection

Participant data will be collected using Computer-Assisted Personal Interview (CAPI) at three time points: baseline (when participants are first enrolled in the study), eight months post-baseline (upon program completion), and 3–4 months following endline data collection. Data collection will take place in a private space and interviews will not proceed if anyone besides the interviewer and participant is present. Data collectors will be trained using a combination of online learning modules and in-person training, which will cover the study tools, evaluation protocol, humanitarian principles and its current practice, referral processes, safeguarding policies, Psychological First Aid (PFA) gender and GBV, and research ethics and best practice. All participants in need of a referral will be linked to the appropriate services by HIAS, according to their case management system and referral database. Referral pathways will be established based on HIAS’ robust networks with other humanitarian organizations in the study sites.

All study procedures were reviewed and approved by the institutional review board (IRB) of Universidad de Los Andes (IRB00007443) in Bogota, Colombia. The approval number is 1863, and the official certification was issued on February 27, 2024.

#### Study outcome measures and ascertainment

Self-reliance: The Self-Reliance Index (SRI) is the first-ever global tool for measuring the progress of refugee households toward self-reliance and is currently being used by over 40 refugee-serving organizations around the globe [26]. The tool contains twelve domains focused on a household’s basic needs, resources, and sustainability. Domains measure conditions and assets that increase the likelihood that refugees will be able to continue meeting their needs in the future. The study participant will respond on behalf of her household. The final self-reliance score may take a value from 1 to 5, with higher scores reflecting greater self-reliance. The tool is available in Spanish and has been previously adapted with Venezuelan migrants in Colombia.

Resilience: The Brief Resilient Coping Scale (BRCS) captures capacities to cope with stress adaptively. The scale focuses on the effective use of coping strategies in a flexible and committed manner to actively solve problems in the face of stressful circumstances [27].

Women’s empowerment: Women’s empowerment will be measured using the Women’s Multidimensional Empowerment Index, which was developed using the 2015 Colombian Demographic and Health Survey [28]. The index assesses empowerment multidimensionally based on the definition of empowerment proposed by Kabeer [29] (Resources, domestic decision making, decision making on personal matters, and achievements), while capturing the notion of disempowerment.

Self-efficacy and social support: Three items on self-efficacy and social support will also be included in the survey questionnaire. These three questions are part of a longer tool HIAS employs with women clients to assess their empowerment and knowledge and perception of GBV risks. This HIAS tool was adapted based on other tools used in the Latin American region. The three items included in this evaluation pertain to self-reported perceptions of feeling supported by the community, feeling useful, and feeling controlled by one or more people. Responses are on a Likert scale. Participants will also be asked whether they have accessed GBV-related services in the last three months.

Mental Health: The Spanish version of the Patient Health Questionnaire-9 (PHQ-9) will be used to measure the prevalence and severity of depressive symptoms in the last two weeks. The PHQ-9 includes 9 items related to recent experiences of common symptoms of depression that are rated on a 4-point Likert scale. This tool has been validated in clinical and non-clinical samples and among Venezuelan immigrants/refugees, as well as in the Colombian population, demonstrating excellent reliability (alpha = 0.90) and validity for a unidimensional model of depression and a bidimensional model (depressive symptoms somatic and affective) [30].

#### Statistical methods

Preliminary and descriptive analyses will include: one-way frequency distributions of each survey item and item composite scores, including measures of central tendency (e.g. mean) and variability (e.g. standard deviation) for continuous, approximately normally distributed composite scores. Cronbach’s alpha of 0.6 and above will be used as a threshold cut-off to determine the reliability of the composite scores.

We will also model the variation in participants’ responses across time. We will test and compute effect sizes for the following hypotheses:

1. Directly following program implementation, women who participate in the intervention will exhibit greater improvement in household self-reliance, mental health, and resilience, compared with women who do not participate in the intervention.
2. 3–4 months following the completion of program implementation, women who participate in the intervention will exhibit greater improvement in household self-reliance, mental health, and resilience, compared with women who do not participate in the intervention.

We will test these hypotheses using random coefficient models. The random coefficient model is similar to conducting a difference in difference analysis, but controls and adjusts for additional covariates such as time, taking into account the fact that the data are repeatedly measured or correlated over time. These models incorporate random intercepts and slopes for each research participant based upon the participant’s multiple measurements on outcomes captured over time (pre-test versus post-test) [31]. All models will include both time-constant demographic (e.g. prior academic achievement and nationality) as well as time-varying antecedent covariates (e.g., phasing of program implementation).

### Generating learning on SRI thresholds

Data will also be collected from approximately 80 participants in a fourth city, Ipiales, though these participants will not be included in the RCT. Data from this group will instead be used to explore current implementation practices and eligibility thresholds. The current practice to only include respondents in the study who score between a 2 and 4 on the SRI was based on discussions between the research and implementation teams around *who* has the capacity to most benefit from ESGL. The teams agreed that households scoring below a certain value on the SRI are likely to be more in need of acute aid, and unlikely to be able to take advantage of livelihoods support. The teams also agreed that households scoring above a certain value are more likely to be sustainably self-reliant and thus not a priority target for the program. Discussions around the appropriate floor and ceiling thresholds were speculative in nature, with the teams ultimately agreeing to define eligibility as scoring between 2 and 4 on the SRI. In order to further inform discussions on appropriate thresholds, participants in Ipiales will be assigned to a study arm based on their SRI score, where those with the lowest scores will be assigned to the treatment arm and those with the highest scores will be in the control group. This data will help us understand whether those with a low SRI score of 2 at baseline can realize significant gains, while also examining whether those who are already nearing self-reliance continue to progress without the aid of programming.

## Discussion

Over the past decade, humanitarian crises have not only grown in scale, affecting more individuals, but have also prolonged in duration. Acknowledging the drawn-out nature of conflicts in recent decades, the humanitarian community has broadened its approach beyond just providing immediate assistance to actively fostering resilience and self-reliance among forcibly displaced populations. This shift in approach is partly in response to the inadequacies of traditional emergency response frameworks, which have inadvertently fostered dependency on aid while diminishing the agency and skills of displaced populations. Strengthening self-reliance is not only empowering but also has the potential to reduce the long-term costs of aid provision, as individuals become more capable of providing for themselves and relying less on external support. This pilot evaluation will help measure the ESGL model to foster the self-reliance and wellbeing of forcibly displaced women in the Colombian context. Examining our primary research hypotheses will elucidate whether an entrepreneurial program for forcibly displaced women at risk of GBV can effectively bolster self-reliance, resilience, and mental health, or whether other programmatic components are needed. In addition to providing important insights about pathways to well-being for forcibly displaced women in the Colombian context, findings will contribute to a larger global evidence base around programming that can promote the self-reliance of displaced women in other contexts where this population is afforded the right-to-work.
